# Supplementary material for: Assessing allocation bias in stratified clinical trials with multi-component endpoints evaluated using the stratified Wei-Lachin test
Source: PLoS One. 2026 Feb 13;21(2):e0341039. doi: 10.1371/journal.pone.0341039 (PMC12904587; doi:10.1371/journal.pone.0341039)
Supplement: S1 Appendix — Explanation of abbreviations and symbols that has been used in the manuscript. (PDF) [file pone.0341039.s001.pdf]

S1 Appendix. Abbrevations and symbols

Table S1.1: Abbreviations used in the main manuscript.

**Abbreviation**	**Description**
EMA	European Medicine Agency
WL	Wei-Lachin
RP	Randomization Procedure
EBC	Efron Biased Coin Design
BSD	Big Stick Design
MP	Maximal Procedure
RAR	Random Allocation Rule
PBR	Permuted Block Randomization
T1E	Type I error
ERDO	Evaluation of Randomization Procedures for Design Optimization
PCOMs	Patient-centered outcome measures
GPC	Generalize pairwise comparison

Table S1.2: Notations and symbols used in the main manuscript.

**Symbol**	**Description**
K	Number of strata
m	Number of endpoint components
$\mathbf{E}$	Experimental group
$\mathbf{C}$	Control group
N	Sample size
n_j	Number of patients in stratum j
$n_{j,E}$	Number of patients in **E** of stratum j
$n_{j,C}$	Number of patients in **C** of stratum j
$t_j = (t_{j,1}, \ldots, t_{j,n_j})^T$	Allocation vector with $t_{j,i} = 1$ ($t_{j,i} = 0$) if patient i in stratum j is allocated to **E** (**C**)
$X_{j,i} = (X_{j,i,1}, \ldots, X_{j,i,m})^T$	Response vector of patient i in stratum j
μ_E	Expected response vector for **E**
μ_C	Expected response vector for **C**
Σ	Common but unknown covariance matrix for **E** and **C**
0_m	m-dimensional vector with only zeros
$\tau_{j,i}$	Allocation bias effect on the responses of patient i in stratum j
$n_{j,E}(i-1)$	Number of allocations to **E** after $i-1$ assignments in stratum j
$n_{j,C}(i-1)$	Number of allocations to **C** after $i-1$ assignments in stratum j
$\eta_j = (\eta_{j,1}, \ldots, \eta_{j,m})$	Biasing factor for stratum j that may vary across endpoint components and strata
$\mathcal{N}_m(0_m, I_m)$	Multivariate standard normally distribution
I_m	Identity matrix of dimension m
n_{sim}	Simulation number
$\Delta_{N,K,m}$	Effect size of the stratified Wei-Lachin test for N patients, K strata and m endpoint components
t_{WL}	Wei-Lachin test statistic
$t''(N-2K, \delta, \lambda)$	Doubly non-central t-distribution with non-centrality parameters δ and λ, and $N-2K$ degrees of freedom
$F(\cdot; N-2K, \delta, \lambda)$	Distribution function of the doubly non-central t-distribution with parameters δ and λ, and $N-2K$ degrees of freedom
$t_{N-2K}(1-\alpha)$	$(1-\alpha)$-quantile of the central t-distribution with $N-2K$ degrees of freedom
d	A given vector and chosen as $(1, \ldots, 1)^T \in \mathbb{R}^m$
D_j	Mean treatment differences of the endpoint components in stratum j given by $\overline{X_{j,E}} - \overline{X_{j,C}}$.
w_j, w_j^*	Weights of the stratified WL test statistic chosen as $\frac{n_{j,E} n_{j,C}}{n_{j,E}+n_{j,C}}$
$\hat{\Sigma}_p$	Pooled covariance matrix
