# Supplementary material for: Assessing allocation bias in stratified clinical trials with multi-component endpoints evaluated using the stratified Wei-Lachin test
Source: PLoS One. 2026 Feb 13;21(2):e0341039. doi: 10.1371/journal.pone.0341039 (PMC12904587; doi:10.1371/journal.pone.0341039)
Supplement: S2 Appendix — Proof that the stratified WL test statistic is doubly non-central t-distributed under biased conditions. (PDF) [file pone.0341039.s002.pdf]

S2 Appendix. Distribution of the stratified WL test statistic under allocation bias

Note: If a reference to an equation is marked with the abbreviation "m.M." it indicates that the reference is to the equation in the main manuscript. For example, (1, m.M.) refers to the first equation in the main manuscript.

Assuming that patient responses are independent and distorted by allocation bias as described by the model (1, m.M.) and the allocation bias policy (4, m.M.) and that the strata are independent, then the stratified WL test statistic

$$t_{WL} = \frac{d^T \sum_{j=1}^K w_j D_j}{\sqrt{\sum_{j=1}^K \frac{w_j^2}{w_j^*}} \sqrt{d^T \hat{\Sigma}_p d}}$$

follows the distribution

$$t_{WL} \sim t''(N - 2K; \delta, \lambda),$$

where $t''(N-2K;\delta,\lambda)$ denotes the doubly non-central t-distribution [1] with $N - 2K$ degrees of freedom and non-centrality parameters δ and λ defined as

$$\delta = \frac{d^T \sum_{j=1}^K w_j (\mu_E - \mu_C + \overline{\tau_{j,E}} - \overline{\tau_{j,C}})}{\sqrt{\sum_{j=1}^K \frac{w_j^2}{w_j^*} d^T \Sigma d}}$$

$$\lambda = \frac{\sum_{j=1}^K \sum_{i=1}^{n_j} (d^T \tau_{j,i})^2 - n_{j,E}(d^T \overline{\tau_{j,E}})^2 - n_{j,C}(d^T \overline{\tau_{j,C}})^2}{d^T \Sigma d}$$

with $\overline{\tau_{j,E}} = \frac{1}{n_{j,E}} \sum_{i=1}^{n_j} \tau_{j,i} t_{j,i}$ and $\overline{\tau_{j,C}} = \frac{1}{n_{j,C}} \sum_{i=1}^{n_j} \tau_{j,i}(1 - t_{j,i})$.

Proof. The proof relies on the proof by Hilgers [2], who showed that the Fleiss test statistic in stratified single endpoint clinical trials follows a doubly non-central t-distribution under allocation bias.
The test statistic of the stratified WL test can be rewritten as:

$$t_{WL} = \frac{\left(d^T \sum_{j=1}^K w_j \sqrt{\sum_{j=1}^K \frac{w_j^2}{w_j^*}}^{-1} (\overline{X_{j,E}} - \overline{X_{j,C}}) \right) / \sqrt{d^T \Sigma d}}{\sqrt{d^T \hat{\Sigma}_p d / \sqrt{d^T \Sigma d}}} := \frac{t_{WL1}}{t_{WL2}}.$$

1. Distribution of the numerator t_{WL1}

Since the patient responses are independent multivariate normally distributed, we have

$$\overline{X_{j,E}} \sim \mathcal{N}_m(\mu_E + \overline{\tau_{j,E}}, \frac{1}{n_{j,E}} \Sigma) \text{ and } \overline{X_{j,C}} \sim \mathcal{N}_m(\mu_C + \overline{\tau_{j,C}}, \frac{1}{n_{j,C}} \Sigma).$$

This implies

$$\frac{d^T \sum_{j=1}^K w_j (\overline{X_{j,E}} - \overline{X_{j,C}})}{\sqrt{\sum_{j=1}^K \frac{w_j^2}{w_j^*}}} \sim \mathcal{N} \left(\frac{d^T \sum_{j=1}^K w_j (\mu_E - \mu_C + \overline{\tau_{j,E}} - \overline{\tau_{j,C}})}{\sqrt{\sum_{j=1}^K \frac{w_j^2}{w_j^*}}}, d^T \Sigma d \right).$$

Thus, the numerator t_{WL1} is normally distributed by

$$t_{WL1} \sim \mathcal{N}\left(\frac{d^T \sum_{j=1}^K w_j(\mu_E - \mu_C + \overline{\tau_{j,E}} - \overline{\tau_{j,C}})}{\sqrt{\sum_{j=1}^K \frac{w_j^2}{w_j^*} d^T \Sigma d}}, 1\right).$$

2. Distribution of the denominator t_{WL2}

Now, we consider

$$\begin{aligned}(N-2K)t_{WL2}^2 =& \frac{1}{d^T \Sigma d}\Bigg(d^T \Bigg[\sum_{j=1}^K \sum_{i=1}^{n_j} (X_{j,i} - \overline{X_{j,E}})(X_{j,i} - \overline{X_{j,E}})^T t_{j,i} \\ &+ (X_{j,i} - \overline{X_{j,C}})(X_{j,i} - \overline{X_{j,C}})^T (1-t_{j,i})\Bigg] d\Bigg) \\ =& \frac{\sum_{j=1}^K \sum_{i=1}^{n_j} (d^T X_{j,i} - d^T \overline{X_{j,E}})^2 t_{j,i} + (d^T X_{j,i} - d^T \overline{X_{j,C}})^2 (1-t_{j,i})}{d^T \Sigma d}\end{aligned}$$

Since $\frac{d^T X_{j,i}}{d^T \Sigma d}$, $1 \leq j \leq K$, $1 \leq i \leq n_j$ are independent normally distributed variables, the terms

$$\sum_{i=1}^{n_j} \frac{(d^T X_{j,i} - d^T \overline{X_{j,E}})^2 t_{j,i}}{d^T \Sigma d} \quad \text{and} \quad \sum_{i=1}^{n_j} \frac{(d^T X_{j,i} - d^T \overline{X_{j,C}})^2 (1-t_{j,i})}{d^T \Sigma d}$$

are χ^2-distributed [3] with $n_{j,E} - 1$ and $n_{j,C} - 1$ degrees of freedom and non-centrality parameters

$$\lambda_{j,E} = \sum_{i=1}^{n_j} \frac{(d^T \tau_{j,i} - d^T \overline{\tau_{j,E}})^2 t_{j,i}}{d^T \Sigma d}, \quad \lambda_{j,C} = \sum_{i=1}^{n_j} \frac{(d^T \tau_{j,i} - d^T \overline{\tau_{j,C}})^2 (1-t_{j,i})}{d^T \Sigma d}.$$

The sum of independent non-central χ^2-distributed variables is also non-central χ^2-distributed [1]. Thus, $(N-2K)t_{WL2}^2$ is non-central χ^2-distributed with non-centrality parameter

$$\sum_{j=1}^K \lambda_{j,E} + \lambda_{j,C} = \frac{1}{d^T \Sigma d} \sum_{j=1}^K \sum_{i=1}^{n_j} (d^T \tau_{j,i})^2 - n_{j,E}(d^T \overline{\tau_{j,E}})^2 - n_{j,C}(d^T \overline{\tau_{j,C}})^2$$

and

$$\sum_{j=1}^K n_{j,E} + n_{j,C} - 2 = N - 2K.$$

degrees of freedom. Altogether, it holds

$$(N-2K)t_{WL2}^2 \sim \chi^2\left(N-2K, \frac{1}{d^T \Sigma d} \sum_{j=1}^K \sum_{i=1}^{n_j} (d^T \tau_{j,i})^2 - n_{j,E}(d^T \overline{\tau_{j,E}})^2 - n_{j,C}(d^T \overline{\tau_{j,C}})^2\right).$$

3. Independence

Finally, we prove the independence of $d^T \sum_{j=1}^K w_j D_j$ and $(N-2K)d^T \hat{\Sigma}_p d$. The latter can be rewritten as

$$(N-2K)d^T \hat{\Sigma}_p d = \sum_{j=1}^K \sum_{i=1}^{n_j} (d^T X_{j,i} - d^T \overline{X_{j,E}})^2 t_{j,i} + (d^T X_{j,i} - d^T \overline{X_{j,C}})^2 (1-t_{j,i}).$$

According to Searle [4], two random variables expressed as x^TAx and Bx with $x \sim \mathcal{N}(\mu, V)$ are independent if $BVA = 0$. Defining $x_j = (d^TX_{j,1}, \cdots, d^TX_{j,n_j})^T$, we have $V_j = d^T\Sigma d I_{n_j}$ with I_{n_j} the identity matrix of dimension n_j. The term $w_jd^TD_j$ can be expressed as B_jx_j, where

$$B_j = w_j(\frac{1}{n_{j,E}}t_j - \frac{1}{n_{j,C}}(1-t_j))^T.$$

Similarly, the term

$$\sum_{i=1}^{n_j}(d^TX_{j,i} - d^T\overline{X_{j,E}})^2t_{j,i} + (d^TX_{j,i} - d^T\overline{X_{j,C}})^2(1-t_{j,i})$$

can be expressed as $x_j^TA_jx_j$, where

$$A_j = I_{n_j} - \frac{1}{n_{j,E}}t_jt_j^T - \frac{1}{n_{j,C}}(1-t_j)(1-t_j)^T.$$

It suffices to show the matrix equation according to Searle only for a specific stratum j, because of the independent strata and the, therefore, implied block structure of the matrices. Since, $B_jV_jA_j = 0$ we proved the independence of $d^T\sum_{j=1}^K w_jD_j$ and $(N-2K)d^T\hat{\Sigma}_pd$.

4. Final Distribution

In summary, we have shown

$$t_{WL} \sim \frac{\mathcal{N}\left(\frac{d^T\sum_{j=1}^K w_j(\mu_E - \mu_C + \overline{\tau_{j,E}} - \overline{\tau_{j,C}})}{\sqrt{\sum_{j=1}^K \frac{w_j^2}{w_j^*}d^T\Sigma d}}, 1\right)}{\sqrt{\frac{\chi^2\left(N-2K, \frac{1}{d^T\Sigma d}\sum_{j=1}^K\sum_{i=1}^{n_j}(d^T\tau_{j,i})^2 - n_{j,E}(d^T\overline{\tau_{j,E}})^2 - n_{j,C}(d^T\overline{\tau_{j,C}})^2\right)}{N-2K}}}.$$

Thus, t_{WL} is doubly non-central t-distributed with $N-2K$ degrees of freedom and non-centrality parameters

$$\delta = \frac{d^T\sum_{j=1}^K w_j(\mu_E - \mu_C + \overline{\tau_{j,E}} - \overline{\tau_{j,C}})}{\sqrt{\sum_{j=1}^K \frac{w_j^2}{w_j^*}d^T\Sigma d}}$$

$$\lambda = \frac{\sum_{j=1}^K\sum_{i=1}^{n_j}(d^T\tau_{j,i})^2 - n_{j,E}(d^T\overline{\tau_{j,E}})^2 - n_{j,C}(d^T\overline{\tau_{j,C}})^2}{d^T\Sigma d}.$$

This concludes the proof. □

References

[1] N.L. Johnson, S. Kotz, and N. Balakrishnan, *Continuous Univariate Distributions*, Volume 2, Wiley Series in Probability and Statistics, Wiley, New York, 1995.

[2] R-D. Hilgers, M. Manolov, N. Heussen, WF. Rosenberger. *Design and analysis of stratified clinical trials in the presence of bias.* Statistical Methods in Medical Research. 2019;29(6):1715-1727.

[3] S. Langer, *The modified distribution of the t-test statistic under the influence of selection bias based on random allocation rule*, Master Thesis, RWTH Aachen University, 2014.

[4] S.R. Searle, *Linear models*, Wiley, New York, 1971.
