# Supplementary material for: Assessing allocation bias in stratified clinical trials with multi-component endpoints evaluated using the stratified Wei-Lachin test
Source: PLoS One. 2026 Feb 13;21(2):e0341039. doi: 10.1371/journal.pone.0341039 (PMC12904587; doi:10.1371/journal.pone.0341039)
Supplement: S3 Appendix — Simulation results of the misspecified T1Es under different biased clinical scenarios, including unbalanced strata and heterogeneous bias effects across endpoints. (PDF) [file pone.0341039.s003.pdf]

S3 Appendix: Additional simulation results on the impact of allocation bias on the stratified Wei-Lachin test decisions

Below, we present the detailed numerical results of the simulation study introduced in Section 'Simulation study'. The study aims to investigate the impact of allocation bias on the inference of the stratified Wei-Lachin test. Therefore, we calculated the mean T1E under misspecification. Note that the numerical values of the mean T1E under misspecification are rounded to the fourth decimal place.

Numerical results of the simulation study (N=16)

Table S3.1: Impact of allocation bias on the mean T1E under misspecification in clinical trials with $N = 16$ patients for different numbers of centers (K), different numbers of endpoints (m) and common allocation bias effects $\eta = \eta_{j,l}$ for all $j \in \{1, \ldots, K\}$ and $l \in \{1, \ldots, m\}$ chosen as proportion $\rho \in \{0.05, 0.1\}$ of the effect sizes $\Delta_{16,2,2} = 0.93$, $\Delta_{16,2,4} = 0.66$, $\Delta_{16,4,2} = 0.97$ and $\Delta_{16,4,4} = 0.69$.

RP	K	m	ρ	T1E under missp. [mean]
BSD(3)	2	2	0.05	0.0553
		4	0.05	0.0553
		2	0.1	0.0609
		4	0.1	0.0609
	4	2	0.05	0.0568
		4	0.05	0.0569
		2	0.1	0.0642
		4	0.1	0.0643
EBC(0.67)	2	2	0.05	0.0577
		4	0.05	0.0577
		2	0.1	0.0661
		4	0.1	0.0661
	4	2	0.05	0.0590
		4	0.05	0.0590
		2	0.1	0.0689
		4	0.1	0.0691

Table S3.1: Impact of allocation bias on the mean T1E under misspecification in clinical trials with $N = 16$ patients for different numbers of centers (K), different numbers of endpoints (m) and common allocation bias effects $\eta = \eta_{j,l}$ for all $j \in \{1, \ldots, K\}$ and $l \in \{1, \ldots, m\}$ chosen as proportion $\rho \in \{0.05, 0.1\}$ of the effect sizes $\Delta_{16,2,2} = 0.93$, $\Delta_{16,2,4} = 0.66$, $\Delta_{16,4,2} = 0.97$ and $\Delta_{16,4,4} = 0.69$.

RP	K	m	ρ	T1E under missp. [mean]
MP(2)	2	2	0.05	0.0603
		4	0.05	0.0603
		2	0.1	0.0719
		4	0.1	0.0720
	4	2	0.05	0.0617
		4	0.05	0.0618
		2	0.1	0.0753
		4	0.1	0.0754
PBR(4)	2	2	0.05	0.0615
		4	0.05	0.0615
		2	0.1	0.0747
		4	0.1	0.0748
	4	2	0.05	0.0617
		4	0.05	0.0618
		2	0.1	0.0753
		4	0.1	0.0755
RAR	2	2	0.05	0.0590
		4	0.05	0.0591
		2	0.1	0.0691
		4	0.1	0.0692
	4	2	0.05	0.0617
		4	0.05	0.0618
		2	0.1	0.0753
		4	0.1	0.0755

Violin plots of the T1E under misspecification for various clinical scenarios with $N = 16$ patients.

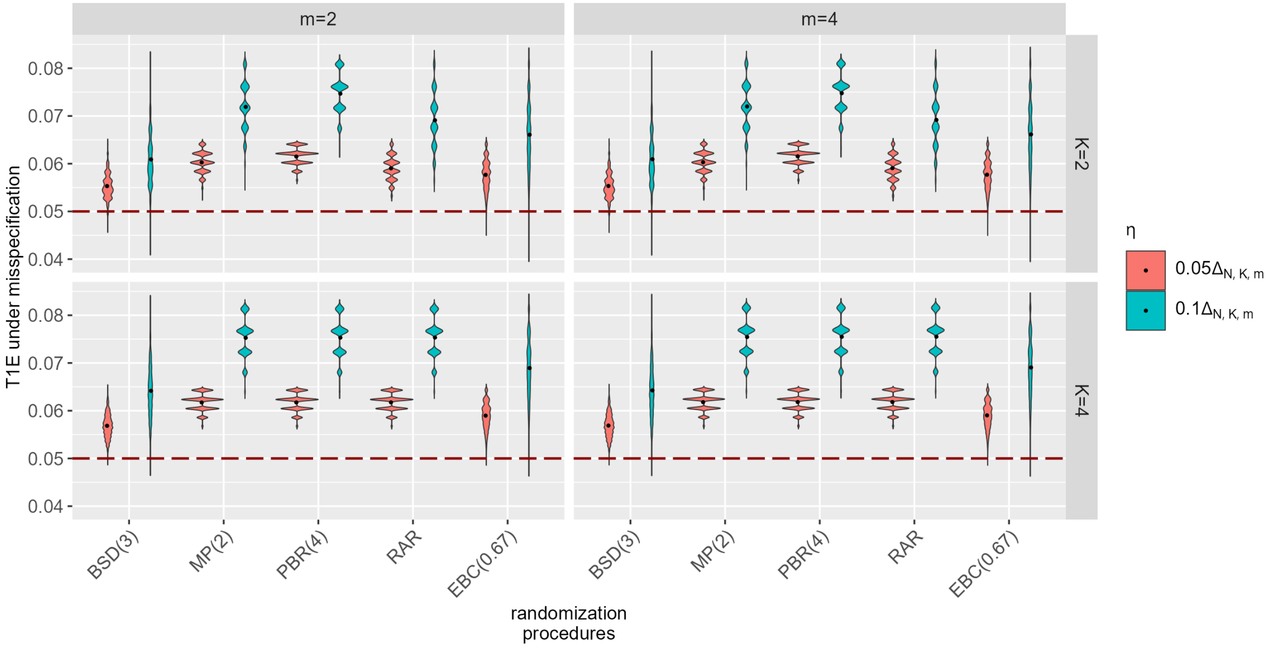

Fig. S3.1: T1Es under misspecification calculated for samples of 10 000 randomization lists generated by different RPs in clinical trials with $N = 16$ patients, K balanced strata, m standard normally distributed uncorrelated endpoints and common allocation bias effect $\eta = \eta_{j,l}$ for all $j \in \{1, \ldots, K\}$ and $l \in \{1, \ldots, m\}$ chosen as proportion $\rho \in \{0.05, 0.1\}$ of the effect sizes $\Delta_{16,2,2} = 0.93$, $\Delta_{16,2,4} = 0.66$, $\Delta_{16,4,2} = 0.97$ and $\Delta_{16,4,4} = 0.69$.

Numerical results of the simulation study (N=32)

Table S3.2: Impact of allocation bias on the mean T1E under misspecification in clinical trials with $N = 32$ patients for different numbers of centers (K), different numbers of endpoints (m) and common allocation bias effects $\eta = \eta_{j,l}$ for all $j \in \{1, \dots, K\}$ and $l \in \{1, \dots, m\}$ chosen as proportion $\rho \in \{0.05, 0.1\}$ of the effect sizes $\Delta_{32,2,2} = 0.64$, $\Delta_{32,2,4} = 0.45$, $\Delta_{32,4,2} = 0.64$ and $\Delta_{32,4,4} = 0.46$.

RP	K	m	ρ	T1E under missp. [mean]
BSD(3)	2	2	0.05	0.0545
		4	0.05	0.0545
		2	0.1	0.0593
		4	0.1	0.0593
	4	2	0.05	0.0554
		4	0.05	0.0555
		2	0.1	0.0611
		4	0.1	0.0613
EBC(0.67)	2	2	0.05	0.0571
		4	0.05	0.0571
		2	0.1	0.0650
		4	0.1	0.0649
	4	2	0.05	0.0578
		4	0.05	0.0579
		2	0.1	0.0663
		4	0.1	0.0666
MP(2)	2	2	0.05	0.0598
		4	0.05	0.0597
		2	0.1	0.0709
		4	0.1	0.0707
	4	2	0.05	0.0604
		4	0.05	0.0605
		2	0.1	0.0722
		4	0.1	0.0726

Table S3.2: Impact of allocation bias on the mean T1E under misspecification in clinical trials with $N = 32$ patients for different numbers of centers (K), different numbers of endpoints (m) and common allocation bias effects $\eta = \eta_{j,l}$ for all $j \in \{1, \ldots, K\}$ and $l \in \{1, \ldots, m\}$ chosen as proportion $\rho \in \{0.05, 0.1\}$ of the effect sizes $\Delta_{32,2,2} = 0.64$, $\Delta_{32,2,4} = 0.45$, $\Delta_{32,4,2} = 0.64$ and $\Delta_{32,4,4} = 0.46$.

RP	K	m	ρ	T1E under missp. [mean]
PBR(4)	2	2	0.05	0.0616
		4	0.05	0.0616
		2	0.1	0.0751
		4	0.1	0.0750
	4	2	0.05	0.0616
		4	0.05	0.0618
		2	0.1	0.0750
		4	0.1	0.0755
RAR	2	2	0.05	0.0569
		4	0.05	0.0569
		2	0.1	0.0644
		4	0.1	0.0643
	4	2	0.05	0.0591
		4	0.05	0.0593
		2	0.1	0.0694
		4	0.1	0.0697

Numerical results of the simulation study (N=64)

Table S3.3: Impact of allocation bias on the mean T1E under misspecification in clinical trials with $N = 64$ patients for different numbers of centers (K), different numbers of endpoints (m) and common allocation bias effects $\eta = \eta_{j,l}$ for all $j \in \{1, \ldots, K\}$ and $l \in \{1, \ldots, m\}$ chosen as proportion $\rho \in \{0.05, 0.1\}$ of the effect sizes $\Delta_{64,2,2} = 0.45$, $\Delta_{64,2,4} = 0.32$, $\Delta_{64,4,2} = 0.45$ and $\Delta_{64,4,4} = 0.32$.

RP	K	m	ρ	T1E under missp. [mean]
BSD(3)	2	2	0.05	0.0543
		4	0.05	0.0543
		2	0.1	0.0589
		4	0.1	0.0590
	4	2	0.05	0.0546
		4	0.05	0.0546
		2	0.1	0.0595
		4	0.1	0.0595
EBC(0.67)	2	2	0.05	0.0569
		4	0.05	0.0570
		2	0.1	0.0646
		4	0.1	0.0647
	4	2	0.05	0.0572
		4	0.05	0.0572
		2	0.1	0.0652
		4	0.1	0.0653
MP(2)	2	2	0.05	0.0596
		4	0.05	0.0596
		2	0.1	0.0704
		4	0.1	0.0706
	4	2	0.05	0.0599
		4	0.05	0.0599
		2	0.1	0.0712
		4	0.1	0.0713

Table S3.3: Impact of allocation bias on the mean T1E under misspecification in clinical trials with $N = 64$ patients for different numbers of centers (K), different numbers of endpoints (m) and common allocation bias effects $\eta = \eta_{j,l}$ for all $j \in \{1, \ldots, K\}$ and $l \in \{1, \ldots, m\}$ chosen as proportion $\rho \in \{0.05, 0.1\}$ of the effect sizes $\Delta_{64,2,2} = 0.45$, $\Delta_{64,2,4} = 0.32$, $\Delta_{64,4,2} = 0.45$ and $\Delta_{64,4,4} = 0.32$.

RP	K	m	ρ	T1E under missp. [mean]
PBR(4)	2	2	0.05	0.0618
		4	0.05	0.0618
		2	0.1	0.0755
		4	0.1	0.0757
	4	2	0.05	0.0618
		4	0.05	0.0618
		2	0.1	0.0755
		4	0.1	0.0757
RAR	2	2	0.05	0.0552
		4	0.05	0.0552
		2	0.1	0.0607
		4	0.1	0.0608
	4	2	0.05	0.0570
		4	0.05	0.0570
		2	0.1	0.0646
		4	0.1	0.0647

Violin plots of the T1E under misspecification for various clinical scenarios with $N = 64$ patients

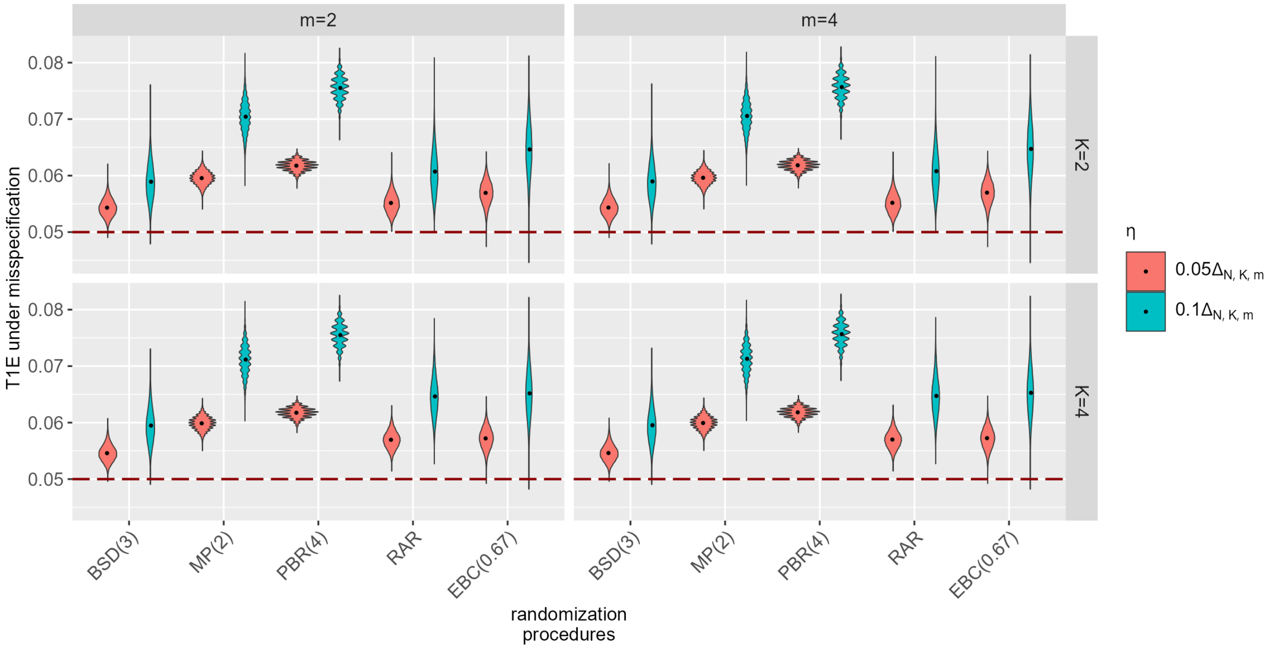

Fig. S3.2: T1Es under misspecification calculated for samples of 10 000 randomization lists generated by different RPs in clinical trials with $N = 64$ patients, K balanced strata, m standard normally distributed uncorrelated endpoints and common allocation bias effect $\eta = \eta_{j,l}$ for all $j \in \{1, \ldots, K\}$ and $l \in \{1, \ldots, m\}$ chosen as proportion $\rho \in \{0.05, 0.1\}$ of the effect sizes $\Delta_{64,2,2} = 0.45$, $\Delta_{64,2,4} = 0.32$, $\Delta_{64,4,2} = 0.45$ and $\Delta_{64,4,4} = 0.32$.

Heterogeneous allocation bias effects

We investigate the impact of heterogeneous allocation bias effects that vary across endpoint components by evaluating the misspecified T1E. The violin plots below illustrate the T1Es under misspecification for different heterogeneous allocation bias effects, based on 10 000 randomization lists generated by various RPs.

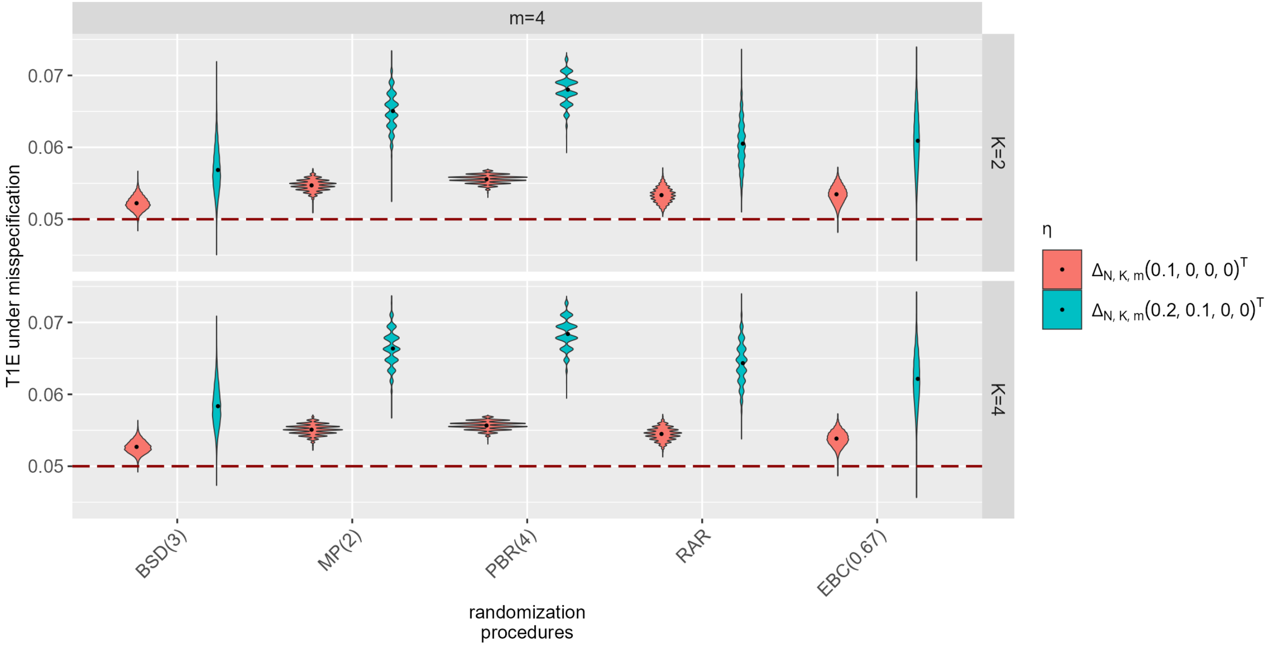

Fig. S3.3: T1Es under misspecification calculated for samples of 10 000 randomization lists generated by different RPs in clinical trials with $N = 32$ patients, K balanced strata, $m = 4$ standard normally distributed uncorrelated endpoints and allocation bias effects $\eta = (\eta_{1,j}, \eta_{2,j}, \eta_{3,j}, \eta_{4,j})$ of $(0.2, 0, 0, 0)\Delta_{32,K,4}$ and $(0.2, 0.1, 0, 0)\Delta_{32,K,4}$ for all $j \in \{1, \dots, K\}$.

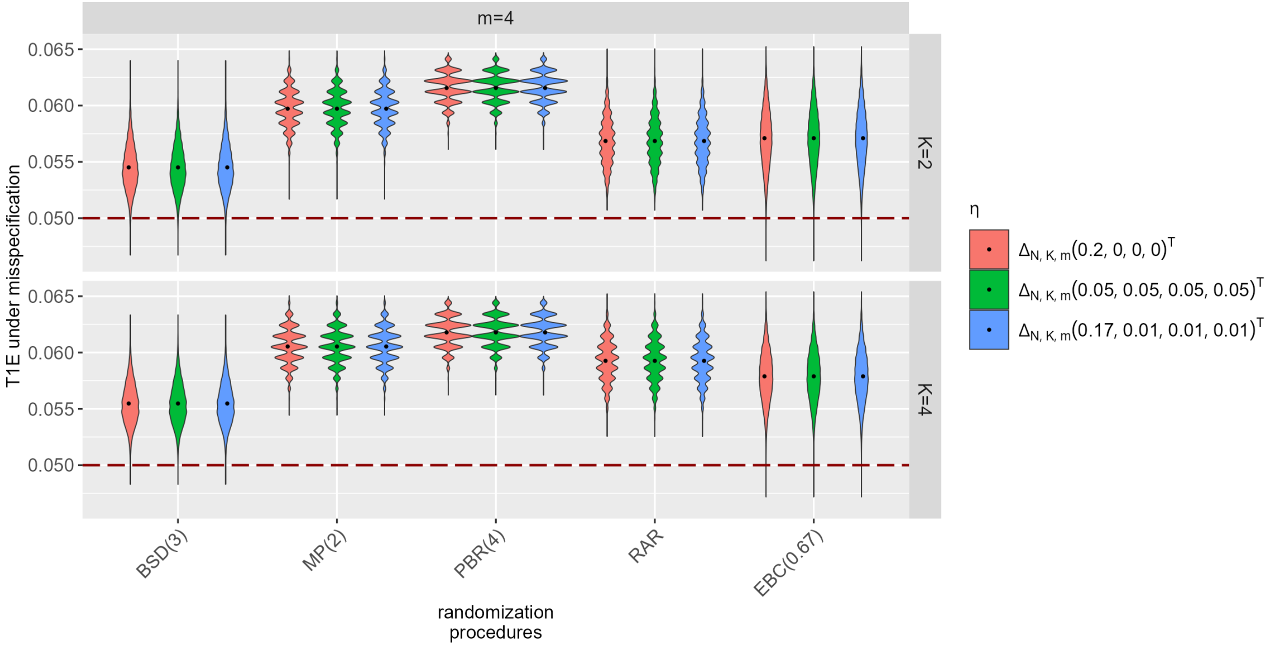

Fig. S3.4: T1Es under misspecification calculated for samples of 10 000 randomization lists generated by different RPs in clinical trials with $N = 32$ patients, K balanced strata, $m = 4$ standard normally distributed uncorrelated endpoints and allocation bias effects $\eta = (\eta_{1,j}, \eta_{2,j}, \eta_{3,j}, \eta_{4,j})$ of $(0.2, 0, 0, 0)\Delta_{32,K,4}$, $(0.05, 0.05, 0.05, 0.05)\Delta_{32,K,4}$ and $(0.17, 0.01, 0.01, 0.01)\Delta_{32,K,4}$ for all $j \in \{1, \dots, K\}$.

Interpretation: When considering heterogeneous allocation bias effects that differ between endpoint components, we observe that a larger sum of endpoint-specific biasing factors results in greater inflation of the T1Es (Fig. **S3.3**). It is negligible whether the bias effect is evenly distributed across all endpoint components or driven by a single component, both scenarios produce the same extent of T1E inflation (Fig. **S3.4**).

Balanced vs imbalanced strata

In the following, we compare the case of balanced and unbalanced strata. We consider trials with $N = 32$ patients, $K = 2$ strata, and $m \in \{2, 4\}$ endpoint components. In the balanced case (1/1), each stratum contains 16 patients, whereas in the unbalanced case (1/3), the first stratum includes 8 patients and the second stratum includes 24 patients.

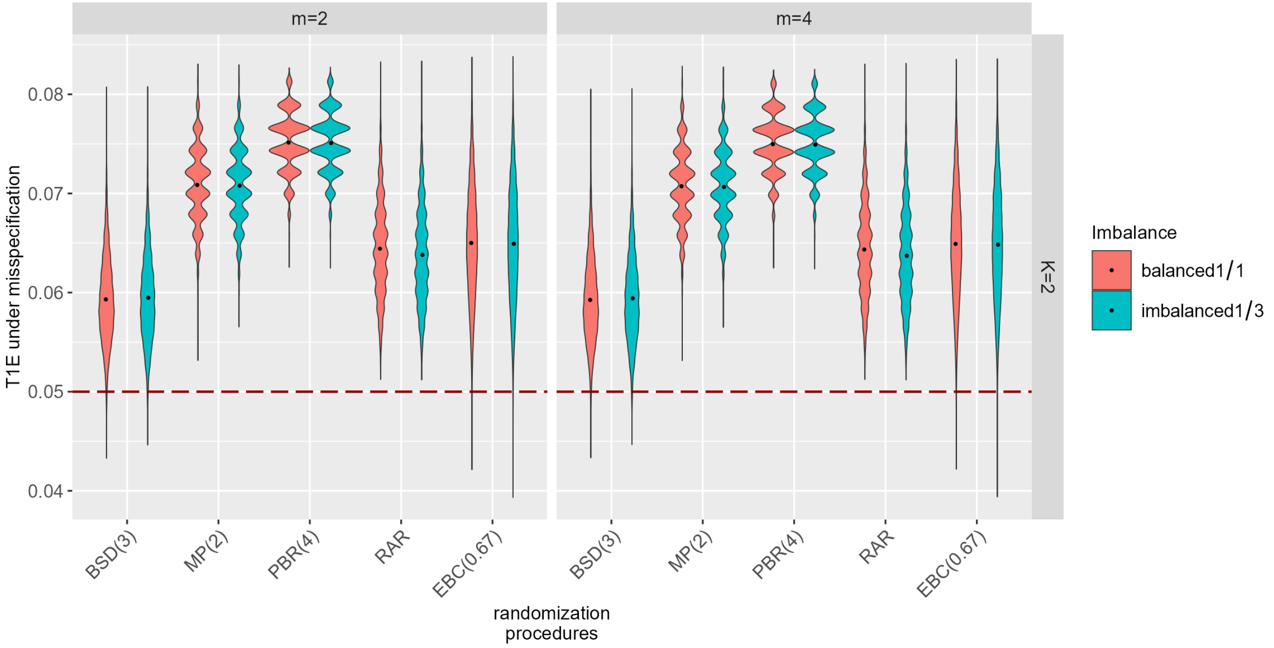

Fig. S3.5: T1Es under misspecification calculated for samples of 10 000 randomization lists generated by different RPs in clinical trials with $N = 32$ patients, K strata that are either balanced (16:16) or unbalanced (8:24), m standard normally distributed uncorrelated endpoints and allocation bias effects $\eta = \eta_{j,l}$ for all $j \in \{1, \ldots, K\}$ and $l \in \{1, \ldots, m\}$ chosen as 10% of the effect sizes $\Delta_{N=32,K=2,m=2} = 0.64$ or $\Delta_{N=32,K=2,m=4} = 0.46$.

Interpretation: The simulation results demonstrate that the impact of allocation bias and the associated T1E inflation in stratified clinical trials with multi-component endpoints depends not on the balancing between strata (Fig. **S3.5**).
