# Supplementary material for: Assessing allocation bias in stratified clinical trials with multi-component endpoints evaluated using the stratified Wei-Lachin test
Source: PLoS One. 2026 Feb 13;21(2):e0341039. doi: 10.1371/journal.pone.0341039 (PMC12904587; doi:10.1371/journal.pone.0341039)
Supplement: S4 Appendix — Comparison of the mean misspecified T1E and family-wise error rates for multiple primary endpoints, co-primary endpoints, and multi-component endpoints, evaluated using the Šidák correction, the all-or-none procedure, and the Wei–Lachin test. (PDF) [file pone.0341039.s004.pdf]

S4 Appendix: Comparison of the impact of allocation bias using different types of multiple endpoints

In the following, we compare the impact of allocation bias in clinical trials with different types of multiple endpoints. We focus on trials with the following types of multiple endpoints:

- **multiple primary endpoints:** Overall efficacy is concluded if at least one endpoint shows a statistically significant treatment effect.
- **co-primary endpoint:** Overall efficacy is concluded if all endpoints show a statistically significant treatment effect.
- **multi-component endpoint:** Different endpoint components are aggregated into a single score or rating that are evaluated for a treatment effect.

To evaluate these types of multiple endpoints, we apply appropriate statistical testing strategies. Multiple primary endpoints are analyzed using the Šidák correction to control the familywise error rate (FWER) at the 5% level. Co-primary endpoints are assessed with an all-or-none decision rule, which ensures control of the type I error rate (T1E) at 5% and multi-component endpoints are evaluated using the Wei–Lachin test.

The impact of allocation bias on the inference of these procedures is assessed by examining the misspecified error rates, which occur when allocation bias is present but ignored during inference. For trials with multiple primary endpoints evaluated using the Šidák correction, we focus on the FWER under misspecification. Whereas, for co-primary endpoints assessed with the all-or-none procedure, and for multi-component endpoints evaluated using the Wei-Lachin test, we assess the T1E under misspecification.

For the theoretical derivation of the evaluation of the impact of allocation bias in trials with multiple primary and co-primary endpoints, we refer to [1].

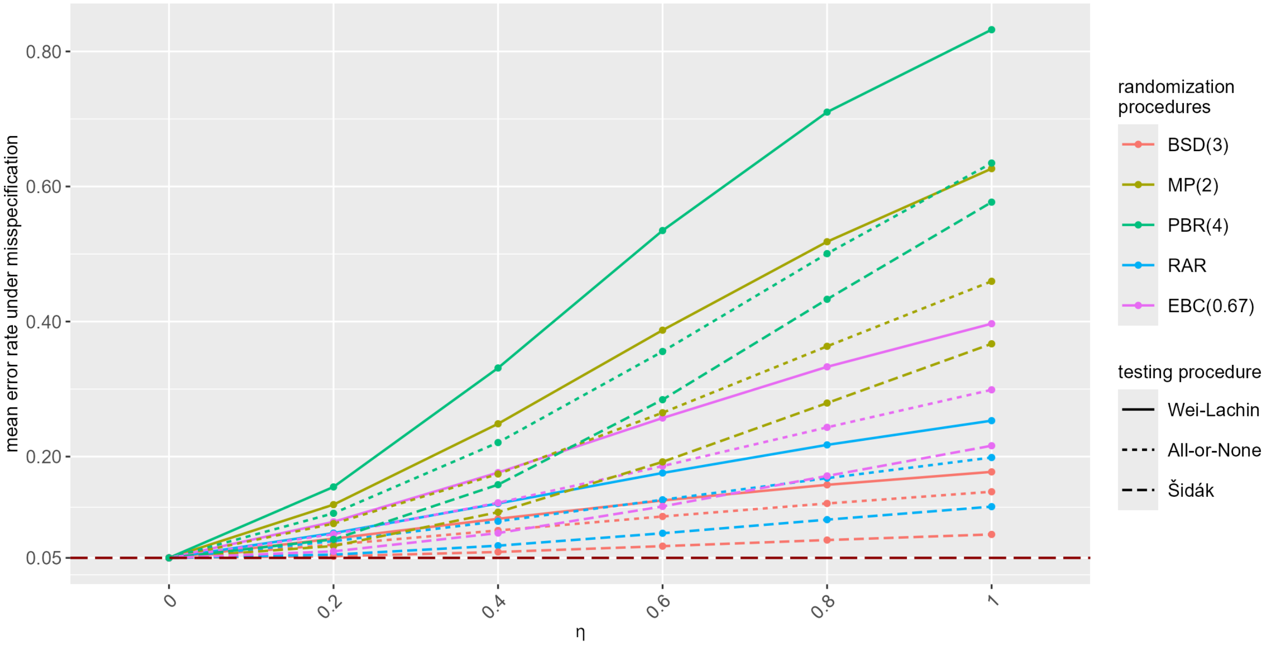

Fig. S4.1: Mean misspecified error rates calculated for samples of 10 000 randomization lists generated by different RPs in clinical trials with $N = 32$ patients, $K = 1$ strata, $m = 2$ uncorrelated normally distributed endpoints and homogeneous allocation bias effects $\eta \in \{0, 0.2, 0.4, 0.6, 0.8, 1\}$ across endpoints. The $m = 2$ endpoints are considered either as multiple primary endpoint evaluated using the Šidák correction, as co-primary endpoint evaluated using the all-or-none procedure or as multi-component endpoint assessed through the WL test.

Interpretation: Fig. **S4.1** shows the mean misspecified error rates for trials with $N = 32$ patients, $m = 2$ endpoints, and homogeneous allocation bias effects $\eta \in \{0, 0.2, 0.4, 0.6, 0.8, 1\}$ across endpoints. Irrespectively of the implemented randomization procedure, allocation bias most strongly inflated error rates when outcomes were combined into a multi-component endpoint and analyzed with the Wei–Lachin test. By contrast, the smallest increases were observed when trials used multiple primary endpoints and applied a Šidák correction. Overall, the influence of allocation bias was substantially less pronounced in trials using multiple primary endpoints than in those relying on multi-component endpoints. Co-primary endpoints evaluated by the all-or-none procedure

were less affected by allocation bias than multi-component endpoints analyzed with the Wei–Lachin test, but they showed greater susceptibility to bias than multiple primary endpoints analyzed with the Šidák correction.

References

[1] Schoenen S, Heussen N, Verbeeck J, Hilgers RD. *The impact of allocation bias on test decisions in clinical trials with multiple endpoints using multiple testing strategies.* BMC Med Res Methodol. 2024 Sep 30;24(1):223. doi: 10.1186/s12874-024-02335-x.
