# Supplementary material for: Assessing allocation bias in stratified clinical trials with multi-component endpoints evaluated using the stratified Wei-Lachin test
Source: PLoS One. 2026 Feb 13;21(2):e0341039. doi: 10.1371/journal.pone.0341039 (PMC12904587; doi:10.1371/journal.pone.0341039)
Supplement: S5 Appendix — Introduction of an approach for bias-adjusted analysis to improve the validity of trials that are potentially affected by allocation bias. (PDF) [file pone.0341039.s005.pdf]

S5 Appendix: Bias-adjusted sensitivity analysis

Note: If a reference to an equation is marked with the abbreviation "m.M." it indicates that the reference is to the equation in the main manuscript. For example, (1, m.M.) refers to the first equation in the main manuscript. We use the same notation as in the main manuscript.

If allocation bias is an issue that may have distorted the trial results, a bias-adjusted sensitivity analysis can be conducted to confirm the results and increase validity.
We consider a stratified clinical trial with a multi-component endpoint, aggregating several endpoint components by a simple sum. We assume that patient responses regarding the multi-component endpoint are affected by allocation bias. The allocation bias effects are modeled using the allocation bias policy introduced in (4,m.M). For the bias-adjusted sensitivity analysis, we set up a regression framework modeling the patient responses regarding the multi-component endpoint, with allocation bias, strata, and treatment effect as fixed effects:

$$d^T X_{j,i} = d^T \mu_E t_{j,i} + d^T \mu_C (1 - t_{j,i}) + \tilde{\gamma} s_{j,i} + \tilde{\eta} b_{j,i} + \epsilon_{j,i}$$

- $d^T \mu_E$ and $d^T \mu_C$ are the expected responses of the treatment and control group regarding the multi-component endpoint
- $\tilde{\gamma}$ is the aggregated strata effect across all endpoint components
- $s_{j,i} = i$ indicates the stratum to which patient j belongs
- $\tilde{\eta}$ denotes the aggregated allocation bias effect across all endpoint components
- $b_{i,j}$ is defined by

$$b_{j,i} = \begin{cases} -1, & \text{if} \quad n_{j,E}(i-1) > n_{j,C}(i-1) \\ 0, & \text{if} \quad n_{j,E}(i-1) = n_{j,C}(i-1) \\ 1, & \text{if} \quad n_{j,E}(i-1) < n_{j,C}(i-1) \end{cases}$$

The regression framework can be rewritten in matrix form as follows:

$$\begin{pmatrix} d^T X_{1,1} \\ \vdots \\ d^T X_{n_1,1} \\ \vdots \\ d^T X_{1,K} \\ \vdots \\ d^T X n_K, K \end{pmatrix} = \begin{pmatrix} 1 & t_{1,1} & s_{1,1} & b_{1,1} \\ \vdots & \vdots & \vdots & \vdots \\ 1 & t_{n_1,1} & s_{n_1,1} & b_{n_1,1} \\ \vdots & \vdots & \vdots & \vdots \\ 1 & t_{1,K} & s_{1,K} & b_{1,K} \\ \vdots & \vdots & \vdots & \vdots \\ 1 & t_{n_K,K} & s_{n_K,K} & b_{n_K,K} \end{pmatrix} \begin{pmatrix} d^T \mu_C \\ d^T \mu_E - d^T \mu_C \\ \tilde{\gamma} \\ \tilde{\eta} \end{pmatrix} + \epsilon,$$

where $\epsilon \sim \mathcal{N}(0_N, \sigma I_N)$.
A global F-test can be used to evaluate test problem (2, m.M) and determine if the expected response vectors of the multi-component endpoint differ between the treatment and control groups after adjusting for allocation bias and stratum effects. In a small simulation study we investigate the performance of this sensitivity analysis regarding their power to detect significant treatment and allocation bias effects. We consider a clinical trial with $N = 32$ patients, $m = 2$ uncorrelated, normally distributed endpoints and $K = 2$ balanced strata. We assume an overall treatment effect on the multi-component endpoint of 0.64. This effect size corresponds to an effect that yields 80% power for the stratified WL test in the unbiased case. We explore scenarios with homogeneous allocation bias effects across endpoint components where the aggregated allocation bias effects affecting the multi-component endpoint are $\tilde{\eta} \in \{0, 0.4, 0.8, 1.2, 1.6, 2\}$. The power of detecting significant treatment and allocation bias effects is simulated by generating first 1000 randomization lists for each randomization procedure and than calculating for each randomization list the power using 2000 simulated clinical trials. The simulations were conducted in R-4.4.3. We used the R package randomizeR to simulate the different randomization lists and the anova() function of the car package for the global F-test.

Fig. **S5.1** shows the mean power of the global F-test across the 1000 simulated randomization lists for each randomization procedure to detect significant treatment and allocation bias effects. We find that the global F-test may be underpowerd as sensitivity analysis to detect true treatment or allocation bias effects. A power of 80% to detect significant allocation bias effects is only achieved for $\tilde{\eta}$ greater than 1.2. Therefore, consistent results from the primary and sensitivity analyses support the study results. However, the analysis may be underpowered to detect true treatment effects; thus inconsistent results are not necessarily a sign of an invalid primary analysis. Overall, the best way to achieve valid trial results and prevent distortion due to allocation bias is through bias-mitigating study planning.

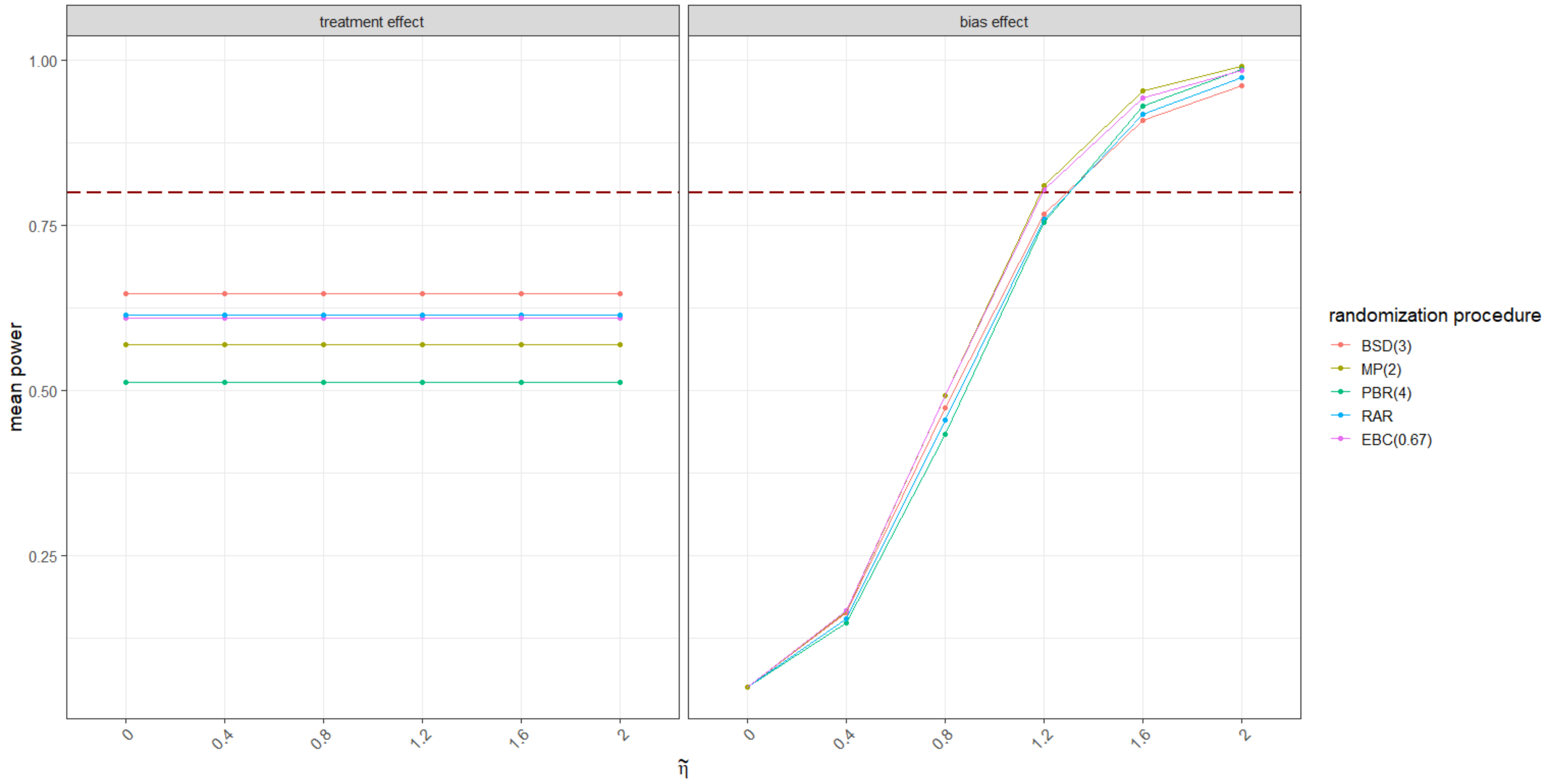

Fig. S5.1: Mean power of the global F-test across the 1000 simulated randomization lists for BSD(3), MP(2), PBR(4), RAR, EBC(0.67) to detect significant treatment and allocation bias effects in the case of $N = 32$ patients, $m = 2$ uncorrelated, normally distributed endpoints and $K = 2$ strata and an assumed overall treatment effect on the multi-component endpoint of 0.64.
