# Supplementary material for: Assessing allocation bias in stratified clinical trials with multi-component endpoints evaluated using the stratified Wei-Lachin test
Source: PLoS One. 2026 Feb 13;21(2):e0341039. doi: 10.1371/journal.pone.0341039 (PMC12904587; doi:10.1371/journal.pone.0341039)
Supplement: Attachment — Submitted filename: Response_to_the_Reviewer.pdf [file pone.0341039.s006.pdf]

Response to the Reviewer

Editor:

This manuscript addresses an important methodological gap in the context of stratified clinical trials with multi-component endpoints, particularly focusing on allocation bias—a critical issue in rare disease trials that often lack blinding. The authors propose a novel allocation biasing policy tailored to such trials and evaluate its impact on the type I error rate of the stratified Wei-Lachin test. The topic is timely, and the methodology appears rigorous.

We thank the editor for this positive assessment.

However, several areas require attention before the manuscript can be considered for publication.
The derivation of the allocation biasing policy is a notable strength. However, the assumptions underlying the model (e.g., independence of endpoint components) should be explicitly stated and justified.

We thank the editor for this suggestion. Independence of the endpoint components is not strictly required for the proposed methodology, as we do not require Σ to be diagonal. However, as usual independence between strata and patients is needed. This assumption is essential for establishing that the test statistic of the stratified Weil–Lachin test follows under the null hypothesis a doubly non-central t-distribution under bias and in the unbiased case the t-distribution. To clarify this point, we have added the following section to the manuscript:

"We consider a randomized, two-arm, parallel-group clinical trial stratified by a fixed factor, e.g. centers or geographical regions, into K independent strata, with a multi-component endpoint that consists of m≥2 not necessarily independent continuous components."

"For independent patients and independent strata, the test statistic t_WL is distributed through [...]"

The simulation study design is comprehensive, but the rationale for selecting specific parameter ranges (e.g., $\rho \in \{0.05, 0.1\}$ should be elaborated upon.

We thank the editor for this valuable comment. We have expanded the manuscript to clarify the rationale behind the parameter choices in our simulation study.

Following common recommendations, we expressed allocation bias as a fraction of the effect size used in the sample size calculation. Choosing 5% and 10% of the effect size allowed us to illustrate that even small to moderate allocation bias effects meaningfully affect the T1E of the stratified WL test. Similar allocation bias effects were chosen in Hilgers et al. (2020, DOI: 10.1177/0962280219846146) and Schoenen et al. (2024, DOI: 10.1186/s12874-024-02335-

x). The evaluation of further allocation bias effects can be conducted using the R Code provided in the repository mentioned in the “Data Availability” statement.

In addition to homogeneous bias scenarios, we also included heterogeneous bias effects across endpoint components. These heterogeneous settings were chosen such that the aggregated bias effect across all endpoint components remained at the same overall level. This allowed us to evaluate whether different endpoint-specific allocation bias patterns, while similar in total, lead to different impacts on test decisions.

We focused on small sample sizes because our goal is to provide planning guidance for improving the validity of rare disease trials, where small samples and multi-component endpoints are common.

The randomization procedures included in the study were selected because they are among the most frequently used procedures in stratified clinical trials. In the manuscript, we further clarify our choices of the parameter settings of the randomization procedures (see p.7).

Furthermore, we examined endpoints with no, moderate, and strong correlations to reflect a broad range of realistic clinical scenarios. We also considered the minimum number of endpoint components and strata required for a study to qualify as a stratified clinical trial with multi-component endpoints (m=2, K=2). Building on this foundation, we then investigated how increasing the number of components or strata influences the extent of allocation bias effects on the test decisions of the stratified WL test.

These clarifications have been added to the manuscript in the section “Simulation study” in the Methods part to make the rationale behind the chosen simulation settings clearer (see p.7).

The finding that the Big Stick Design minimizes type I error inflation is compelling. However, the implications of these findings for practical trial design could be discussed more thoroughly.

We thank the editor for this helpful comment to improve the article. Our simulation study shows that in stratified clinical trials with multi-component endpoints, the Big Stick Design minimize the T1E inflation when allocation bias is a potential issue.

To reduce the impact of allocation bias and ensure valid trial results, future randomized trials should use stratified randomization procedures that allow for limited imbalance of allocations to treatment groups, as within the Big Stick Design. The Big Stick Design is known to reduce the predictability of upcoming assignments.

Informing the scientific community that the Big Stick Design outperforms Permuted Block Randomization in preventing allocation bias effects on test decisions is particularly important, especially given that the randomization section of the ICH E9 guideline (ICH E9,1998) still refers to block randomization. Using Big Stick Design instead of Permuted Block Randomization enhances the reliability and validity of studies that are susceptible to allocation bias.

To determine the most appropriate randomization procedure and their parameter settings for a specific study setting that use multi-component endpoints, we recommend conducting a simulation study during the planning phase using the ERDO framework.

To underline this, we adopted following section in the “Discussion”:

“The strongest inflation occurs when stratified PBR is used and the least inflation when BSD is implemented. The more restrictive the RP, the greater the inflation. Similar findings have been shown in other studies examining allocation bias in other clinical settings, such as in stratified trials with single endpoints [10] or trials with time-to-event data [30]. In stratified clinical trials the most common RP is stratified PBR, however, as the simulations has been shown, these trials are potentially affected by allocation bias, especially when there is a lack of blinding [22]. For future trial designs, we recommend stratified randomization procedures that allow for restricted imbalances between the treatment groups, such as the BSD, to reduce the predictability of upcoming assignments. This will reduce the impact of allocation bias on test decisions and improve trial validity. To determine the most appropriate randomization procedure and their parameter settings for a specific study design, we further advise conducting a simulation study during the planning phase that follows the ERDO framework [14].”

References:

ICH E9. Statistical principles for clinical trials; 1998. Available from: https://www.ema.europa.eu/en/ich-e9-statistical-principles-clinical-trials-scientific-guideline

The manuscript briefly mentions limitations but does not adequately address how these might affect the generalizability of the results.

We thank the editor for this helpful suggestion and agree that the methodology's generalizability is not clearly addressed.

Our results focus on quantifying the effects of allocation bias in clinical trials with small sample sizes and continuous multi-component endpoints evaluated using the stratified WL test. For trials using non-continuous endpoints, more complex testing procedures or multi-component endpoints that are aggregated differently the results may differ. Other randomization procedures or trial designs may be more effective at mitigating allocation bias. For more complex testing procedures or alternative ways of aggregating multi-component endpoints, this framework is a starting point for further generalization as described in the following sections in the ‘Discussion’:

“Further research may extend the methodology to quantify allocation bias to more complex testing procedures, such as the Läuter test, or to test procedures that also can handle non-continuous endpoint types, as the original version of stratified WL test. However, the issue of how to derive the distribution of this test under biased conditions remains unresolved in these cases. To analyze the impact of allocation bias on test decisions of these more advanced test procedures and determine a bias-mitigating trial design, the T1E under misspecification for each randomization list needs to be simulated using a sample of different clinical trials, which becomes computationally demanding. Nevertheless, the proposed allocation bias policy remains applicable even in these more complex settings. Extending the methodology to mixed endpoint types, including binary, time-to-event and continuous data, are necessary for broader applicability, particularly in the context of patient-centered outcome measures [30]. Generalized pairwise comparison (GPC) offer a flexible approach for analyzing multi-component endpoints consisting of several endpoint types [5,35]. The analysis of allocation bias in the context of the GPC statistic and more complex multi-component endpoints is part of future research [35] and

require the extension of the biasing policy to binary endpoints, while for the continuous and time-to-event components the existing biasing policies can be used [14,32].”

Although the allocation bias quantification in the simulation study was performed under specific conditions, our findings suggest that key design aspects, such as the chosen randomization procedure, the number of endpoints, and the stratification scheme, can affect trial validity, particularly in settings that are more susceptible to allocation bias due to missing blinding. These design aspects should therefore be carefully considered during the planning phase of a clinical study. We are aware that the simulation study does not mirror all possible trial scenarios. However, the R code in the repository mentioned in the “Data Availability” statement can be used to evaluate the impact of allocation bias for further trial settings. This fact regarding generalizability is also mentioned in the "Discussion":

“The simulation study focused on analyzing the effects of allocation bias in a few clinical scenarios involving continuous multi-component endpoints, common allocation bias effects across strata, balanced strata, restricted stratified RPs, small sample sizes, and homogeneous weights across strata and endpoints. The simulations demonstrate that design components such as the number of endpoint components, the number of strata, and the randomization procedure influence the impact of allocation bias on the stratified WL test decision. These aspects should be carefully considered during the planning phase of a clinical trial. To determine a bias-mitigating design for a specific clinical scenario, a tailored simulation study reflecting the study's particular settings and evaluating the relevant design components using the outlined framework and R code may be necessary.”

Furthermore, we expanded the discussion on whether the finding that the Big Stick Design reduces allocation bias can be generalized to other clinical scenarios. This result is consistent with findings from allocation bias quantification in other trial settings and supports the recommendation to use randomization procedures that allow for some imbalance between treatment groups, such as the Big Stick Design, to mitigate potential allocation bias. In the ‘Discussion’ section, we adapted the following to illustrate this:

“For future trial designs, we recommend stratified randomization procedures that allow for restricted imbalances between the treatment groups, such as the BSD, to reduce the predictability of upcoming assignments. This will reduce the impact of allocation bias on test decisions and improve trial validity.”

Reviewer 1

However, the manuscript would be strengthened by a more detailed explanation or a reference for the "doubly non-central t-distribution" of the test statistic under bias, as this is a key technical point that may be unfamiliar to many readers.

Thank you very much for your insightful comment. You are right that recognizing that the test statistic under allocation bias follows a doubly non-central t-distribution is one of the essential technical steps required to quantify the impact of allocation bias on the test decisions of the stratified Wei-Lachin test. For the formal definition and a comprehensive overview of the properties of the doubly non-central t-distribution, we refer the reader to Johnson, Kotz, and Balakrishnan. The doubly non-central t-distributed random variable X with non-centrality parameters δ and λ is given by

$$X = \frac{Z + \delta}{\sqrt{\frac{W}{v}}},$$

where Z is standard normally distributed and W follows a chi-squared distribution with v degrees of freedom and non-centrality parameter λ.

We have revised the manuscript accordingly so that readers are directly provided with a suitable reference for further details on this distribution.

"For the definition and a comprehensive review of the properties of the doubly non-central t-distribution, we refer to Johnson, Kotz, and Balakrishnan [26, p.213]"

A recommended addition would be to briefly suggest potential methods for the proposed "bias-adjusted sensitivity analysis using a regression framework," as this is a valuable recommendation for practitioners but is currently mentioned without detail.

Thank you very much for your valuable suggestion. We have now added a brief methodological description of the proposed bias-adjusted sensitivity analysis using a regression framework to the Appendix (S5 Appendix). In addition, we have included a small simulation study to evaluate the performance of this approach. In the revised 'Discussion' section, we now explicitly refer readers to S5 Appendix for a more detailed description of the bias-adjusted sensitivity analysis.

Reviewer 2

A few minor observations: what future research would be needed to continue the study, and what are its limitations?

We thank the reviewer for this question. Further research is needed to investigate allocation bias effects in trials using more complex forms of multi-component endpoints, rather than aggregating the endpoint components into a single composite score. In general, the proposed procedure for quantifying allocation bias effects is transferable to more complex aggregated multi-component endpoints. However, in such cases, determining the distribution of the test statistic under allocation bias becomes more challenging. To address this, the T1E for a specific randomization list can be estimated using a set of simulated clinical trials. This approach can be computationally demanding.

The analysis of allocation bias effects in trials with mixed multi-component endpoints, consisting of continuous, time-to-event, and binary components, is also of considerable interest, as such endpoints are frequently discussed in rare disease research in the context of patient-centered outcome measures. To quantify allocation bias in this setting the allocation bias policy needs to be extended to binary endpoints. For continuous and time-to-event endpoints existing biasing policies can be used (Rückbeil et al.,2017; Hilgers et al., 2017).

Overall, the introduced methodology is a valuable tool for enhancing the evidential strength and validity of clinical trials by supporting the design of trials that mitigate bias. Although the simulation study was conducted under specific conditions, it shows that aspects of the study design, such as the randomization procedure, number of endpoints and strata, can affect trial validity. This is particularly the case in trials that are more susceptible to allocation bias, such as open-label studies. Consequently, design aspects such as the randomization procedure chosen, the number and type of endpoints, and stratification should be carefully considered during the planning phase. Ideally, the specification of such design aspects should be guided by simulation studies to ensure the implementation of a bias-mitigating trial.

The new open science requirements in the updated CONSORT guideline create future opportunities to evaluate what are reasonable allocation bias effects in clinical trials.

We will emphasize these aspects more clearly in the 'Discussion' section of the manuscript to give readers a clearer insight into future investigations and possible issues with these investigations:

"Further research may extend the methodology to quantify allocation bias to more complex testing procedures, such as the Läuter test, or to test procedures that also can handle non-continuous endpoint types, as the original version of stratified WL test. However, the issue of how to derive the distribution of this test under biased conditions remains unresolved in these cases. To analyze the impact of allocation bias on test decisions of these more advanced test procedures and determine a bias-mitigating trial design, the T1E under misspecification for each randomization list needs to be simulated using a sample of different clinical trials, which becomes computationally demanding. Nevertheless, the proposed allocation bias policy remains applicable even in these more complex settings. Extending the methodology to mixed endpoint types, including binary, time-to-event and continuous data, are necessary for broader applicability, particularly in the context of patient-centered outcome measures [30]. Generalized pairwise comparison (GPC) offer a flexible approach for analyzing multi-component endpoints consisting of several endpoint types [5,35]. The analysis of allocation bias in the context of the GPC statistic and more complex multi-component endpoints is part of future research [35] and require the extension of the biasing policy to binary endpoints, while for the continuous and time-to-event components the existing biasing policies can be used [14,32]. In the future, the selection of a clinical trial design should also consider the potential impact of other types of bias, such as time trend, to ensure that the selected trial design mitigate bias best and yield the most reliable results [14]. The new open science requirements in the updated CONSORT guideline 2025 [36] create future opportunities to evaluate what are reasonable allocation bias effects in clinical trials. These kinds of questions will be addressed within RealiseD, a project funded under the EU Innovative Health Initiative public-private partnership framework [37]."

References:

Rückbeil MV, Hilgers RD, Heussen N. Assessing the impact of selection bias on test decisions in trials with a time-to-event outcome. Stat Med. 2017 Jul 30;36(17):2656-2668. doi: 10.1002/sim.7299. Epub 2017 Apr 17. PMID: 28417471; PMCID: PMC5516162.

Hilgers RD, Uschner D, Rosenberger WF, Heussen N. ERDO - a framework to select an appropriate randomization procedure for clinical trials. BMC Med Res Methodol. 2017 Dec 4;17(1):159. doi: 10.1186/s12874-017-0428-z. PMID: 29202708; PMCID: PMC5715815.

In the methods section, support the methodology used with a citation.

We thank the reviewer for this suggestion. We have strengthened the methodological justification by adding further citations. First, we refer to studies that have quantified allocation bias using similar approaches in other study designs. The following passage has been added to the 'Methods' section:

"The approach for quantifying allocation bias is guided by studies that have analyzed allocation bias in other study designs, such as parallel-group single endpoint trials [14] and single endpoint trials with stratified randomization [10]."

In addition, we provide further references to support the choice of our simulation settings. These have been added in the section 'Simulation study':

"We simulate the misspecified T1Es concerning various RPs in different clinical scenarios, summarized in Table 1. Thereby, we focus on RPs that are most commonly used in stratified clinical trials [25]. The parameters of the RPs are chosen as follows: for EBC, we set $p = 2$, as recommended in [16]; for BSD, we use $a = 3$, following [26]; for PBR, we choose $k = 4$ as the largest block size that can be applied uniformly across the examined sample sizes $N \in \{12, 32, 64\}$; and for MP we choose $a = 2$ as an example of a small maximum tolerated imbalance. We restrict the simulations to clinical trials with small sample sizes, reflecting our goal of providing design and planning guidance for more valid rare disease trials. We consider the minimum number of endpoint components and strata required for a study to qualify as a stratified clinical trial with multi-component endpoint ($m = 2$, $K = 2$). Building on this foundation, we then investigate how increasing the number of endpoint components or strata influences the extent of allocation bias effects. Further, we examine endpoints with no, moderate, and strong correlations to reflect a broad range of realistic clinical scenarios. Following prevalent recommendations, we select the allocation bias effect as fraction of the effect size [14]. Thus, we analyze the common allocation bias effect $\eta_{j,l} = \eta$ for all $j \in \{1, . . . , K\}$ and $l \in \{1, . . . , m\}$ selected as fraction $\rho \in \{0.05, 0.1\}$ of the effect size $\Delta_{N,K,m}$ that corresponds to the overall effect size across the multiple outcomes combined in the multi-component endpoint. This allows us to determine if small to moderate allocation bias effects meaningfully impact the T1E of the stratified WL test."

Finally, the derivation of the doubly non-central t-distribution of the stratified Wei–Lachin test statistic under bias is supported by an additional reference that formally defines the doubly non-central t-distribution and describes its key properties.

"For the definition and a comprehensive review of the properties of the doubly non-central t-distribution, we refer to Johnson, Kotz, and Balakrishnan [26, p.213]"

Further foundational literature on the methodology is cited, including the original publication of the Wei–Lachin test, Fleiss's work on stratified analysis, and the guessing strategy used in the allocation bias policy of Blackwell and Hodges.

There are other studies that have been conducted in previous years. In this case, indicate what is new in this section (just answer; do not add to the manuscript).

Thank you for this question, which we appreciate to address in more detail. In previous years, studies on the impact of allocation bias have already been conducted in other study designs, such as single endpoint trials (Hilgers et al., 2017; Tamm et al., 2012; Kennes et al., 2011), multi-arm trials (Diane et al.,2018), trials with time-to-event data (Rückbeil et al.,2017; Rückbeil et al., 2018) . To our knowledge, this is the first study that investigate the effects of allocation bias and the choice of randomization procedures specifically in stratified clinical trials with multiple endpoints that are aggregated into a single multi-component outcome. This topic is particularly relevant for rare disease trials, where evidence is often sparse, open-label designs render trials more susceptible to allocation bias and stratification is often to be assumed promising. Assessing the impact of allocation bias in the design phase of a clinical trial is crucial for choosing an optimal trial design, including the choice of randomization procedure, the number of endpoint components and the number of strata, increasing evidence generated from this trial and assess the value of stratification and the use of multi-component endpoints.

Before our investigation, it was unclear whether findings generalize to stratified trials with multi-component endpoints and thus being able to compare stratified and unstratified designs with multi-component endpoints regarding their ability to prevent allocation bias. Further one could hypothesize that, in more complex trial settings the effect of allocation bias diminishes to the point where it becomes negligible.

To investigate this hypothesis, we develop a methodology to quantify allocation bias in stratified clinical trials with multi-component endpoints analyzed using the stratified Wei-Lachin test. In a simulation study, we identify design settings, including randomization procedure, number of strata and endpoints, that effectively prevent distortion of trial results due to allocation bias. We find that the Big Stick Design, offering also in this setting strong protection against allocation bias across strata.

Finally, our results emphasize an important implication for the development of new patient-centered outcome measures, particularly in rare diseases (Morel et al., 2017). While it may be tempting to include many components to capture a broad spectrum of patient experience, the number of components should be no larger than necessary as each endpoint component may contribute to the overall allocation bias effect.

Furthermore, it has been demonstrated that in small sample trials with multi-component endpoints, including too many strata can increase the impact of allocation bias on test decision. Therefore, when designing a clinical trial, it is important to ensure that the number of patients per stratum should not be smaller than the total number of strata.

We hope that these clarifications make clear which methodological contributions and findings of our work are novel in comparison with previous research.

References:

Kennes LN, Cramer E, Hilgers RD, Heussen N. The impact of selection bias on test decisions in randomized clinical trials. Stat Med. 2011 Sep 20;30(21):2573-81. doi: 10.1002/sim.4279. Epub 2011 Jun 30. PMID: 21717489.

Tamm M, Cramer E, Kennes LN, Heussen N. Influence of selection bias on the test decision. A simulation study. Methods Inf Med. 2012;51(2):138-43. doi: 10.3414/ME11-01-0043. Epub 2011 Nov 21. Erratum in: Methods Inf Med. 2014;53(5):343. PMID: 22101391.

Rückbeil MV, Hilgers RD, Heussen N. Randomization in survival studies: An evaluation method that takes into account selection and chronological bias. PLoS One. 2019 Jun 3;14(6):e0217946. doi: 10.1371/journal.pone.0217946. PMID: 31158260; PMCID: PMC6546249.

Rückbeil MV, Hilgers RD, Heussen N. Assessing the impact of selection bias on test decisions in trials with a time-to-event outcome. Stat Med. 2017 Jul 30;36(17):2656-2668. doi: 10.1002/sim.7299. Epub 2017 Apr 17. PMID: 28417471; PMCID: PMC5516162.

Hilgers RD, Uschner D, Rosenberger WF, Heussen N. ERDO - a framework to select an appropriate randomization procedure for clinical trials. BMC Med Res Methodol. 2017 Dec 4;17(1):159. doi: 10.1186/s12874-017-0428-z. PMID: 29202708; PMCID: PMC5715815.

Morel T, Cano SJ. Measuring what matters to rare disease patients - reflections on the work by the IRDiRC taskforce on patient-centered outcome measures. Orphanet J Rare Dis. 2017 Nov 2;12(1):171. doi: 10.1186/s13023-017-0718-x. PMID: 29096663; PMCID: PMC5667521.

Uschner D, Hilgers RD, Heussen N. The impact of selection bias in randomized multi-arm parallel group clinical trials. PLoS One. 2018 Jan 31;13(1):e0192065. doi: 10.1371/journal.pone.0192065. PMID: 29385190; PMCID: PMC5792025.
